# IAGRN: An Interleaved-Attention Graph Neural Network for Gene Regulatory Network Inference

**DOI:** 10.3390/ijms27167300

**Published:** 2026-08-16

**Authors:** Yue Wang, Sicheng Tian, Dan Li

**Affiliations:** School of Computer Science and Artificial Intelligence, Northeast Forestry University, Harbin 150040, China; wangyue77@nefu.edu.cn (Y.W.); sichengtian@nefu.edu.cn (S.T.)

**Keywords:** gene regulatory networks, single-cell RNA sequencing, graph neural networks, attention mechanisms, deep learning frameworks

## Abstract

Gene regulatory networks (GRNs) describe regulatory interactions between transcription factors and their target genes and are essential for understanding cellular processes and disease mechanisms. Recent advances in single-cell RNA sequencing (scRNA-seq) have enabled data-driven GRN inference at single-cell resolution. However, the high sparsity and noise inherent in scRNA-seq data pose substantial challenges for accurately recovering regulatory relationships. Existing graph neural network (GNN)-based approaches often rely on localized message passing, which can lead to over-smoothing and limited modeling of long-range regulatory dependencies. To address these limitations, a structure-aware interleaved-attention graph learning framework, termed IAGRN, is proposed for GRN inference from scRNA-seq data. Specifically, it interleaves topology-constrained local attention with distance-aware global attention, enabling effective integration of structural priors and long-range regulatory signals. Graph Laplacian positional encoding is further incorporated to preserve topological information and enhance node representations. Evaluations on seven public benchmark datasets demonstrate that IAGRN consistently improves GRN reconstruction under highly sparse conditions and achieves competitive performance compared with existing approaches.

## 1. Introduction

Gene regulatory networks (GRNs) characterize regulatory interactions between transcription factors (TFs) and their target genes (TGs) and play a central role in controlling cellular processes, tissue development, and responses to environmental stimuli [1]. Accurate reconstruction of GRNs is therefore essential for understanding how regulatory programs govern cell fate decisions and how their dysregulation contributes to the development of complex diseases, including cancer, autoimmune disorders, and inherited genetic disorders [2]. Experimental approaches for identifying gene regulatory interactions, such as chromatin immunoprecipitation and perturbation-based assays, provide valuable biological insights but are often labor-intensive, costly, and difficult to scale to genome-wide analyses. Consequently, computational inference of GRNs from high-throughput transcriptomic data has become an important strategy for systematically investigating gene regulatory mechanisms.

Recent advances in single-cell RNA sequencing (scRNA-seq) have enabled transcriptome profiling at single-cell resolution, providing unprecedented opportunities to study gene regulation in heterogeneous cellular populations [3]. Compared with conventional bulk RNA sequencing, scRNA-seq facilitates the characterization of cellular heterogeneity, reconstruction of developmental trajectories, and identification of cell-type-specific regulatory programs [4]. Importantly, inferring GRNs from scRNA-seq data allows regulatory relationships to be examined within individual cellular states, thereby enabling the discovery of dynamic regulatory mechanisms that are often obscured in population-averaged measurements. Such cell-resolved regulatory networks provide valuable insights into lineage commitment, cellular differentiation, and disease progression. However, scRNA-seq data are characterized by high dimensionality and substantial sparsity, largely arising from technical dropout events in which lowly expressed genes are recorded as zero values [5]. These characteristics complicate the reconstruction of robust and biologically meaningful GRNs from noisy expression profiles.

Despite these challenges, numerous computational methods have been developed for GRN inference. Early machine learning approaches, such as GENIE3 [6] and GRNBoost2 [7], primarily utilize ensemble learning strategies to infer regulatory relationships from gene expression data. Although these methods demonstrate good scalability, they rely heavily on statistical associations and are highly sensitive to the inherent sparsity and high noise levels of scRNA-seq data. To capture more complex regulatory relationships, deep learning approaches such as CNNC [8] and DeepDRIM [9] have been subsequently proposed. While they improve the modeling of nonlinear gene dependencies, these methods often require substantial computational resources and offer limited biological interpretability [10]. Furthermore, mapping regulatory networks into Euclidean grid structures may compromise the intrinsic topological features of gene regulatory systems.

Given that GRNs are intrinsically graph-structured data, graph neural networks, which have also been applied to other biomedical association-prediction tasks [11], have emerged as a natural framework for modeling regulatory interactions. Recent representative works, such as GENELink [12] and GNNLink [13], have successfully formulated GRN inference as a link prediction task. For instance, GNNLink utilizes a graph convolutional network (GCN)-based interaction encoder to extract features, ultimately accomplishing the inference through matrix completion. To further optimize inference efficiency and reduce false positives caused by indirect interactions, GMFGRN [14] constructs a gene-cell heterogeneous graph combined with matrix factorization, significantly reducing computational overhead. Meanwhile, considering the limited generalization ability of fixed GNN architectures across different scRNA-seq datasets, AutoGRN [15] introduces neural architecture search technology to automatically design the optimal graph network structure.

Beyond architectural optimizations, recent studies have begun to explore the topological characteristics and structural properties of gene regulatory networks. For example, LineGRN [16] introduces line graph neural networks to directly model gene pairs, alleviating the information propagation bottleneck induced by the disproportionate influence of low-degree nodes. To preserve the directed structure within regulatory cascades, DGCGRN [17] employs directed graph convolutional networks. In a similar vein, GAEDGRN [18] integrates a gravity-inspired graph autoencoder with PageRank-based gene importance scoring to prioritize key regulatory genes.

Concurrently, another line of research focuses on enhancing local feature extraction capabilities and mitigating the over-smoothing problem common in GNNs. Specifically, GATCL [19] incorporates convolutional operations to improve computational efficiency. GeneLink+ [20] adopts the GATv2 [21] architecture to refine dynamic attention allocation, while GRANet [22] integrates residual mechanisms with convolutional feature extraction to strengthen local representation learning.

Specifically, existing GNN-based models still face two major challenges in GRN inference. First, most of them heavily rely on a predefined gene regulatory topology and perform information propagation only between directly connected genes. In practice, however, the initial network is often incomplete because of experimental noise, data sparsity, or limited prior biological knowledge. As a result, some true regulatory relationships may be missing from the input graph. Under such circumstances, models that strictly follow the given topology have limited ability to discover these hidden regulatory links, making it difficult to correct false-negative edges in the network. Second, gene regulation is not always limited to direct interactions. Many regulatory effects are transmitted through intermediate genes and form long-range cascades across the regulatory system. Conventional local message passing mainly captures neighborhood-level information and lacks a global view of the entire gene network. Simply stacking more GNN layers to enlarge the receptive field may cause gene representations to become overly similar, weakening gene-specific regulatory features and reducing the model’s ability to identify complex regulatory relationships. Therefore, a key challenge in GRN inference is how to preserve meaningful biological topology while going beyond local neighborhoods to capture potential long-range regulatory dependencies.

**Figure 1:**
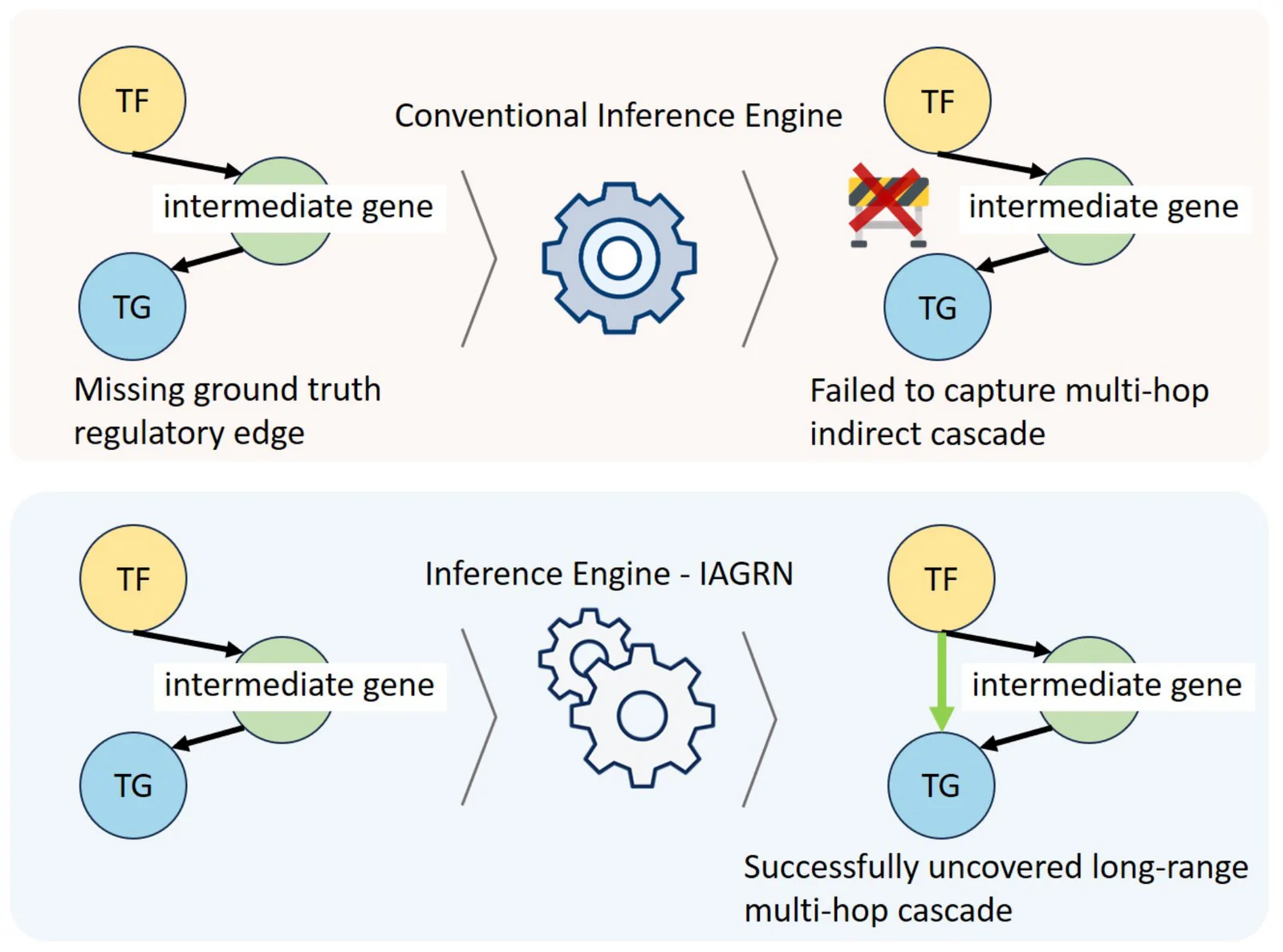
Conceptual comparison between a local message-passing GNN and IAGRN. The red cross indicates a candidate regulatory link not captured in the illustrated local-only setting, whereas the green arrow indicates a nonlocal link captured through global information exchange in IAGRN. Black arrows denote observed local connections through an intermediate gene. This schematic is conceptual and does not represent quantitative results.

To address this challenge, we propose IAGRN, an interleaved-attention graph learning framework for GRN inference. As conceptually illustrated in Figure 1, the central idea of IAGRN is to reconcile topology-guided local learning with global regulatory dependency modeling within a unified attention-based architecture. Rather than being confined to conventional local message passing, the encoder alternates between topology-masked attention and graph positional encoding-enhanced global self-attention. Specifically, topology-masked attention incorporates the known regulatory network as a structural prior, enabling the model to preserve biologically meaningful local constraints and emphasize reliable interactions among neighboring genes. In parallel, global self-attention establishes information exchange across the entire gene system, allowing the model to capture long-range regulatory cascades and uncover potential interactions absent from the initial topology. Through this interleaved design, IAGRN preserves the advantages of topology-aware representation learning while enhancing robustness to incomplete and noisy input networks. By relaxing the strict locality constraint of message passing, IAGRN mitigates over-smoothing and learns more discriminative representations for complex gene regulatory relationships.

In summary, the main contributions of this work are as follows:

- We propose IAGRN, an interleaved-attention graph learning framework for gene regulatory network inference from scRNA-seq data, which integrates local topological structure and global regulatory dependencies within a unified graph learning architecture.
- We develop a structure-aware attention mechanism that combines topology-constrained attention, global self-attention, and graph Laplacian positional encoding, enabling effective information propagation beyond local neighborhoods while preserving biologically meaningful topological information.
- We evaluated IAGRN for GRN inference using seven public scRNA-seq benchmark datasets. The results demonstrate its robustness, yielding better performance than baseline methods in highly sparse conditions.

## 2. Results

### 2.1. Datasets

We evaluated the proposed method on seven scRNA-seq datasets curated in the BEELINE benchmark suite [23], enabling systematic assessment of model performance across diverse biological contexts. These datasets span two species (human and mouse) and capture diverse biological processes, including pluripotency, hepatocyte differentiation, immune activation, and hematopoietic lineage commitment.

Specifically, the benchmark includes human embryonic stem cells (hESC) [24], human hepatocyte differentiation (hHEP) [25], mouse dendritic cells (mDC) [26], mouse embryonic stem cells (mESC) [27], and three lineage-specific populations derived from mouse hematopoietic stem and progenitor cell differentiation: the erythroid (mHSC-E), granulocyte-monocyte (mHSC-GM), and lymphoid (mHSC-L) lineages [28]. Several of these datasets characterize cellular transitions and lineage differentiation processes, providing challenging scenarios for GRN inference. For each dataset, multiple reference networks are employed as evaluation benchmarks, including functional association networks obtained from STRING [29], non-specific ChIP-seq networks [30], and cell-type-specific ChIP-seq networks [31].

Following the standard preprocessing pipeline recommended by BEELINE [23], genes with zero expression were removed, and analyses were restricted to transcription factor–target gene regulatory relationships. Highly variable genes were selected according to the benchmark protocol, and only regulatory interactions among the retained genes were considered. “TFs+500” contains all transcription factors retained after preprocessing together with 500 highly variable non-TF genes, whereas “TFs+1000” contains the same TF set together with 1000 highly variable non-TF genes. The numbers 500 and 1000 are benchmark-defined feature-scale settings used to examine the effect of the number of candidate target genes on GRN inference performance. The seven benchmark datasets were derived from five GEO-accessioned scRNA-seq studies, with accession identifiers GSE81252 (hHEP), GSE75748 (hESC), GSE98664 (mESC), GSE48968 (mDC), and GSE81682 (mHSC-E, mHSC-GM, and mHSC-L). Detailed dataset statistics are summarized in Table 1.

### 2.2. Experiment Setup

Considering the substantial heterogeneity in sparsity and connectivity patterns across benchmark networks, we adopt differentiated evaluation protocols based on network density characteristics.

For the STRING and Non-specific networks, the ground-truth networks are partitioned into training, validation, and test sets with a ratio of 3:1:1. During training, to alleviate class imbalance and improve representation learning stability, the positive-to-negative sample ratio is strictly balanced at 1:1. In contrast, to better reflect realistic biological scenarios, the test set preserves the original class distribution without artificial balancing. Specifically, the ratio between positive and negative samples is determined according to the network density:   

(1) $$\frac{\mathrm{Positive}}{\mathrm{Negative}}=\frac{\mathrm{NetworkDensity}}{1-\mathrm{NetworkDensity}}.$$

For the Cell-type-specific ground-truth networks, which exhibit highly concentrated active TFs and limited sample size, a more conservative data split is adopted. The dataset is divided into training, validation, and test sets with proportions of 67%, 10%, and 23%, respectively. In this setting, the positive-to-negative sample ratio is strictly maintained at 1:1 across training, validation, and testing phases.

This standardized partitioning strategy ensures consistent evaluation across networks with varying structural properties and facilitates fair cross-dataset comparison.

Within this evaluation protocol, the graph supplied to the encoder is constructed exclusively from positive edges in the training partition and therefore constitutes a training-derived structural prior rather than an independent external biological network. Since the reference edges are partitioned before graph construction, held-out positive interactions from the validation and test partitions do not contribute to the structural information used for representation learning. Validation labels are used only for model selection, whereas test labels are reserved for final performance evaluation. As the node set and expression matrix are shared across partitions, the evaluation is node-transductive and assesses generalization to held-out regulatory interactions.

All model training and evaluation experiments were conducted using an NVIDIA GeForce RTX 2080 Ti GPU with CUDA version 11.8.

### 2.3. Performance Evaluation

We formulate GRN inference as a binary classification task, where each gene pair (i,j) is assigned a label indicating the presence (1) or absence (0) of a regulatory interaction.

Due to the intrinsic sparsity of biological networks, true regulatory interactions account for only a small fraction of all possible gene pairs. Therefore, overall accuracy is not an appropriate performance indicator. Instead, we adopt two threshold-independent metrics for comprehensive evaluation: the area under the receiver operating characteristic curve (AUROC) and the area under the precision–recall curve (AUPRC).

During evaluation, the predicted regulatory probabilities are compared against ground-truth labels obtained from STRING or ChIP-seq databases. Both AUROC and AUPRC are computed on each test dataset. To reduce the impact of stochasticity and ensure the robustness of the experimental evaluation, all subsequent experiments were conducted over 20 independent runs, and the mean value of each metric was reported as the final result.

### 2.4. Experiment Results

The effectiveness of IAGRN for gene regulatory network inference was evaluated by comparing its performance with seven established baseline methods across seven benchmark datasets. The selected baselines encompass a broad range of computational paradigms, including the classical method GENIE3, the deep learning framework DeepSEM [32] and the network embedding model GNE [33], alongside recent GNN-based approaches such as GRANet, GNNLink, GATCL, and GENELink. Experiments were conducted under two gene-scale settings, TFs+500 and TFs+1000, to examine the adaptability of the proposed model under different feature dimensions.

**Figure 2:**
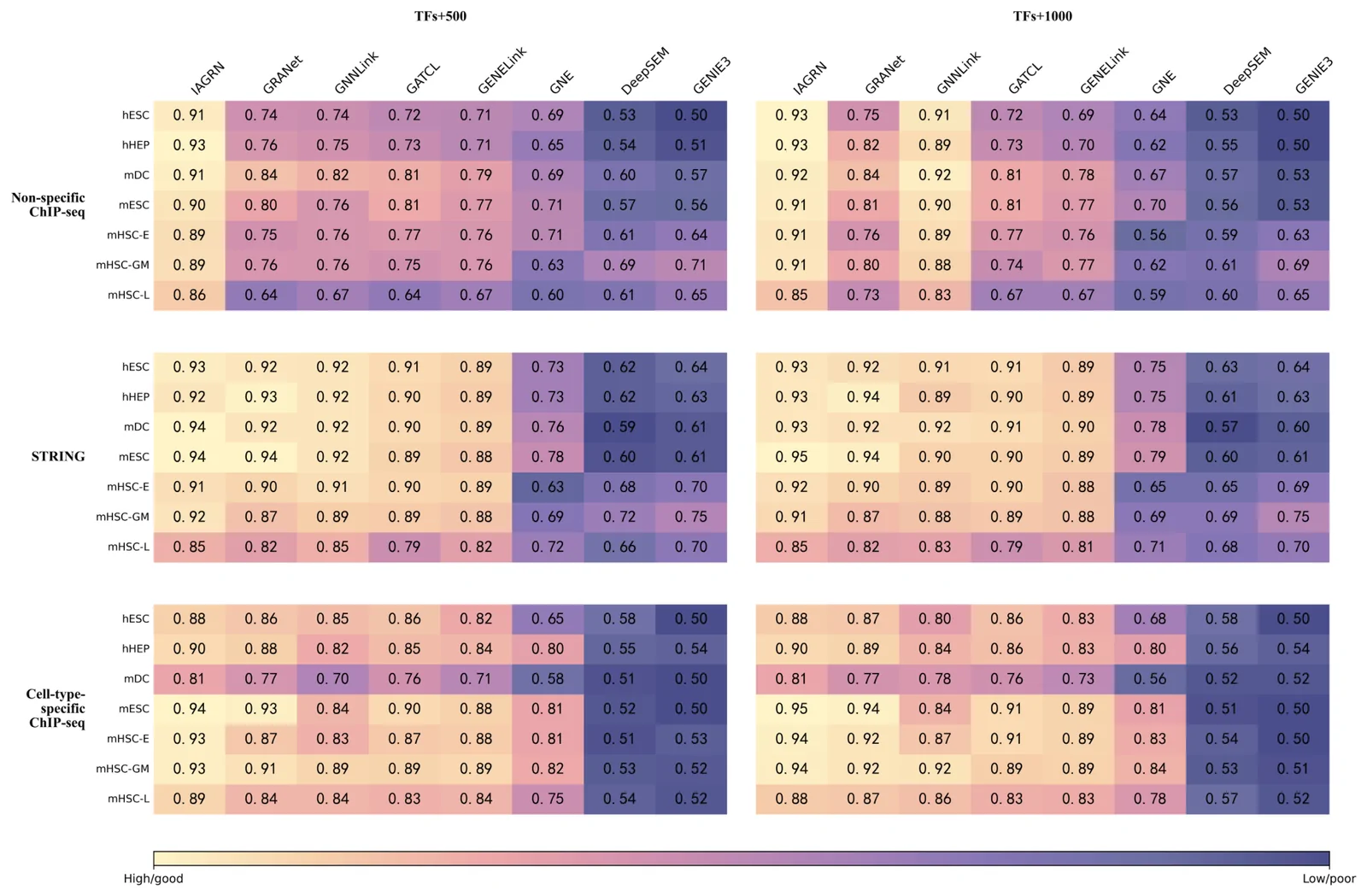
Performance comparison of IAGRN and seven baseline methods across seven benchmark datasets. The heatmap presents AUROC scores under two gene-scale settings: TFs+500 and TFs+1000. Each row corresponds to a specific dataset, and each column represents an inference method. The color gradient indicates model performance, where yellow denotes higher AUROC scores and dark blue denotes lower scores.

As illustrated in Figure 2, in terms of overall predictive performance across all 42 evaluation scenarios (comprising various network sparsities, species, and cell types), IAGRN demonstrates highly competitive and consistent results. Regarding the AUROC metric, IAGRN achieves the highest or joint-highest performance on 39 of the 42 evaluation settings, yielding an average relative improvement of approximately 4.5% over the second-best methods. Notably, IAGRN consistently outperforms both GNN-based architectures (e.g., GRANet, GENELink, GNNLink) and unsupervised approaches (DeepSEM, GENIE3) on the highly noisy and challenging Non-specific ChIP-seq networks. These results support the competitive performance of IAGRN across the evaluated datasets, reference-network types, and gene-scale settings.

**Figure 3:**
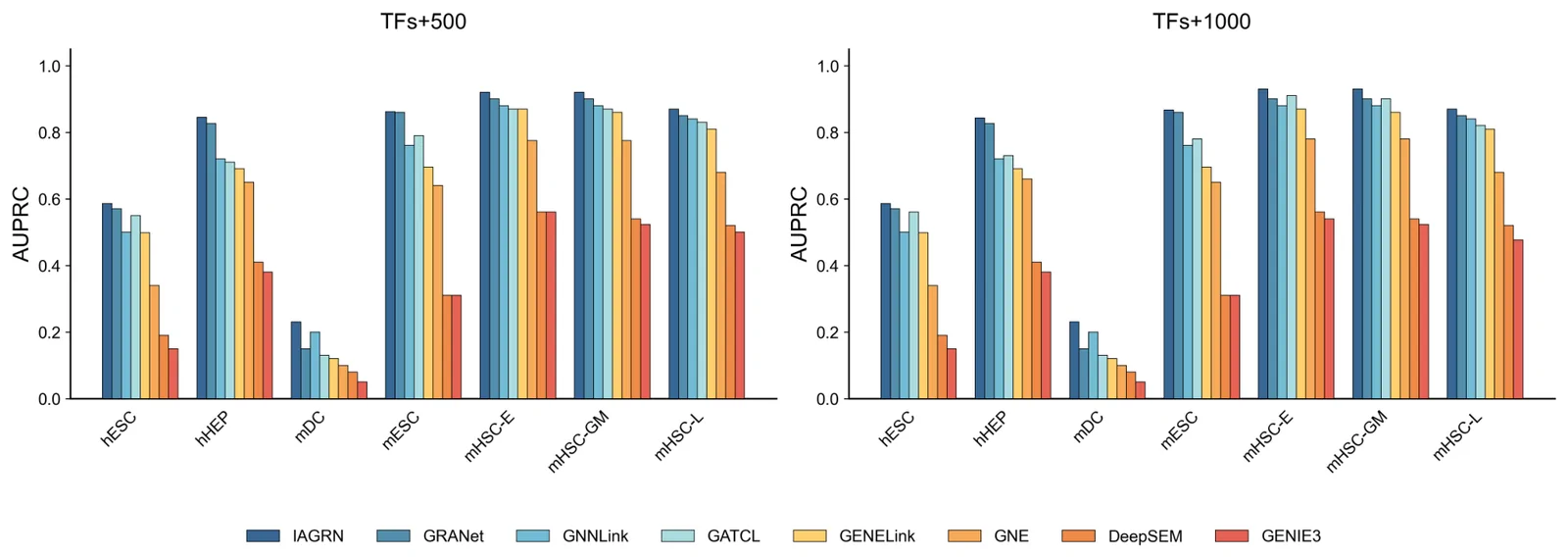
Average AUPRC of eight models on seven single-cell datasets under TFs+500 and TFs+1000 settings, comparing precision–recall trade-offs between IAGRN and baseline methods.

To provide a more comprehensive assessment, particularly considering the extreme sparsity and severe class imbalance inherent in true biological regulatory networks, the evaluation results based on the AUPRC metric are further detailed in Figure 3. Across all 14 evaluated scenarios within the Cell-type-specific ChIP-seq networks, IAGRN consistently yields the highest AUPRC scores, indicating a stable generalization capacity under varying biological conditions. Quantitatively, the proposed framework demonstrates average relative improvements of 3.73%, 9.48%, 9.71%, 15.44%, and 31.97% over GRANet, GATCL, GNNLink, GENELink, and GNE, respectively. Furthermore, when compared to unsupervised or shallow deep learning baselines such as DeepSEM and GENIE3, IAGRN exhibits more pronounced performance gains of 100.91% and 113.93%.

Beyond the macro-level metrics, focusing specifically on the highly noisy Non-specific ChIP-seq group under the TFs+1000 setting reveals how the model attains these favorable results. For example, on the mDC dataset, IAGRN demonstrates clear improvements over existing baselines, outperforming GRANet and GENELink by relative improvements of approximately 8.8% and 17.0% in AUROC, respectively. These findings suggest that combining topology-masked attention with global self-attention may help the model utilize training-derived structural information while reducing its reliance on individual local connections, thereby contributing to the observed improvements in AUROC.

Despite the strong performance of convolution-based baselines like GRANet on STRING and Cell-type-specific networks, IAGRN maintains a consistent advantage. For instance, on the mHSC-E (TFs+1000) dataset, IAGRN surpasses GRANet and GENELink by 2.0% and 4.7% in AUROC, respectively, with similar trends in AUPRC. Unlike convolutional architectures constrained by local receptive fields, IAGRN employs global self-attention to efficiently capture multi-hop regulatory cascades, ensuring robust inference even in highly modular networks.

Furthermore, comparing the TFs+500 and TFs+1000 settings highlights IAGRN’s structural scalability. When target genes double on the mDC dataset, IAGRN’s performance remains remarkably stable across both metrics. Conversely, frameworks like DeepSEM suffer a 5.0% AUROC drop due to the amplified sparsity of the expanded feature space. IAGRN mitigates this dimensionality curse through its adaptive attention modules, which effectively filter out background noise to focus on informative regulatory signals, thereby preserving its performance advantage in large-scale inferences.

### 2.5. Ablation Study

To further investigate the contribution of different modules to the overall performance of IAGRN, we conducted a series of ablation experiments by progressively removing or replacing key components of the model and analyzing the resulting performance variations. Specifically, four model variants were constructed to examine the contributions of global self-attention (GSA), topology-masked attention (TMA), the interleaved stacking strategy, and Laplacian positional encoding (LapPE), as shown in Table 2: (1) w/o GSA: removing the global self-attention module from the encoder, relying solely on masked local attention for feature aggregation; (2) w/o TMA: removing the topology-masked attention module and relying only on purely data-driven global attention; (3) w/o Interleaved: discarding the interleaved stacking strategy and adopting a sequential stacking of local and global modules; (4) w/o LapPE: removing the graph Laplacian positional encoding at the input stage. Each variant was trained using the same parameter settings as the full IAGRN model. Ablation experiments were conducted on seven representative cell-type datasets, and the results were obtained under the same experimental settings as the full model.

**Figure 4:**
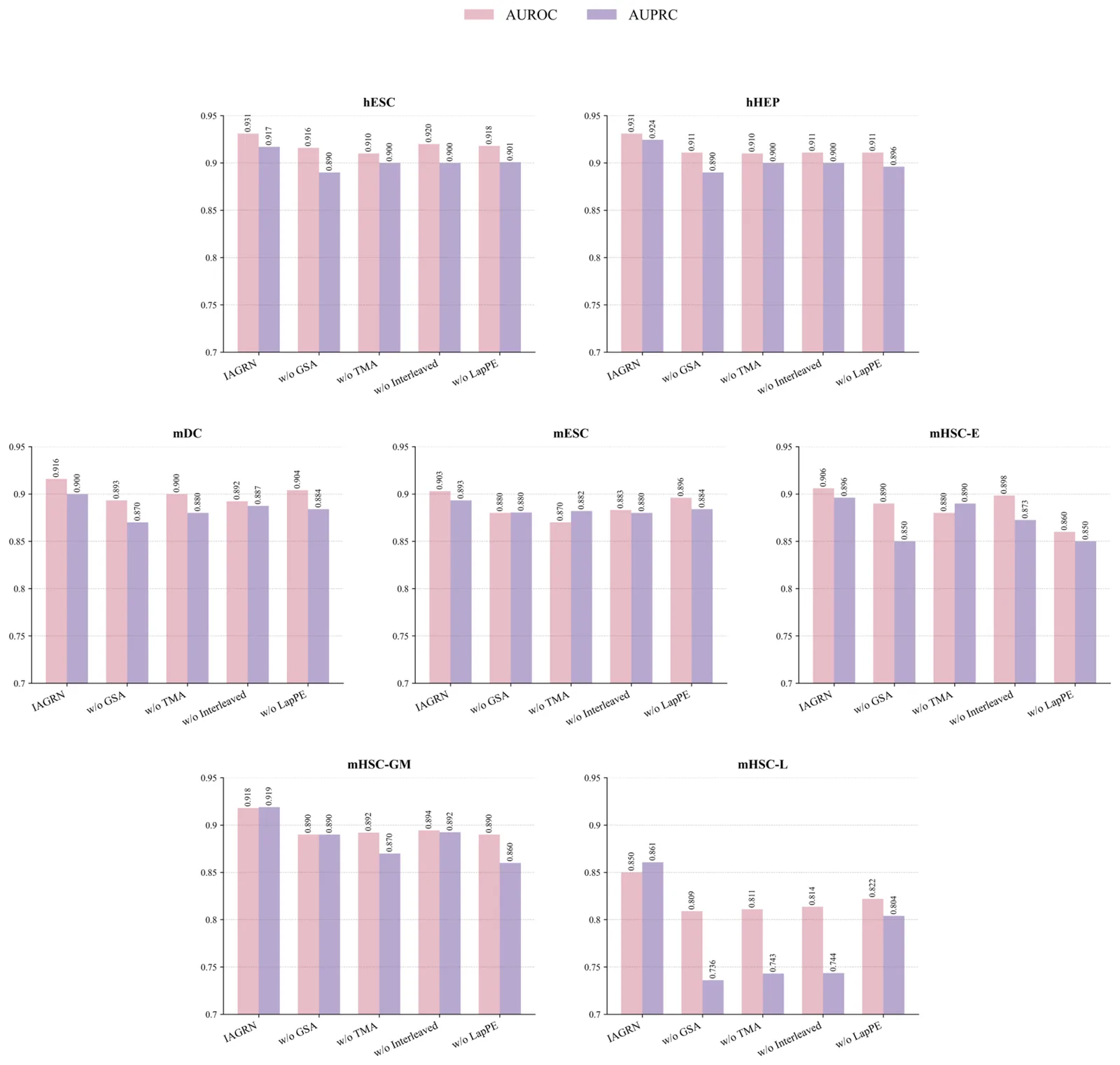
Ablation study results of IAGRN on seven benchmark datasets.

As illustrated in Figure 4, removing any key component leads to varying degrees of performance degradation, supporting the contribution of each component to the overall performance of IAGRN. Notably, disrupting the dynamic balance between global exploration and local constraints can substantially affect model performance. The removal of the global self-attention module (w/o GSA) results in the largest observed decrease: on the mHSC-L dataset, which features complex differentiation trajectories, the AUROC decreases by 4.1%, and the AUPRC decreases by 12.5%. This confirms that relying solely on local aggregation is insufficient for capturing long-range regulatory dependencies. Conversely, removing the local masking constraint (w/o TMA) reduces the AUPRC to 0.743 on the same dataset, indicating that without the structural inductive bias provided by prior networks, the model becomes more susceptible to noise in heterogeneous biological data. Furthermore, altering the network’s alternating design (w/o Interleaved) yields an AUROC decrease of approximately 2.4% on the mDC dataset. Together, these results demonstrate that the interleaved stacking strategy is not merely an architectural choice but an important component for harmonizing local topological constraints with global contextual exploration.

The lower AUPRC observed for mHSC-L may be partly attributable to its more limited labeled interaction set. Under the TFs+1000 setting, mHSC-L contains 847 cells, 692 retained genes, 16 annotated TFs, and 5180 cell-type-specific positive regulatory interactions, compared with 1071 cells, 1204 retained genes, 33 annotated TFs, and 21,975 positive interactions in mHSC-E. This narrower TF coverage and smaller number of labeled interactions may increase evaluation variability and make lymphoid-lineage-specific regulatory links more difficult to distinguish. The larger performance decrease observed after removing GSA further suggests that global contextual information may help compensate for the limited local regulatory evidence available in this data-limited setting. These observations indicate that the lower AUPRC is influenced by the greater difficulty of the mHSC-L evaluation setting and should not be interpreted in isolation as a general limitation of the model.

Beyond the attention architecture itself, the ablation results highlight the important role of topological positional awareness. Removing the Laplacian Positional Encoding (w/o LapPE) reduces the model’s ability to distinguish the topological roles of genes, causing a particularly pronounced AUPRC decrease of 4.6% on the mHSC-E dataset. Mechanistically, this comparatively larger effect may be related to the distinct topology of the mHSC-E prior regulatory network. The mHSC-E prior network may depend more strongly on hierarchical and modular structural cues associated with hub transcription factors. Because global self-attention does not explicitly encode graph topology in the absence of positional information, it may have limited ability to distinguish central hub regulators from peripheral target genes based solely on expression features. By explicitly injecting LapPE, the model encodes the relative topological coordinates of these nodes, preserving hierarchical structural information. Therefore, removing LapPE removes these explicit positional cues, leading to a greater performance reduction in highly modular networks. Ultimately, these ablation results support the contribution of interleaved attention, structural inductive bias, and topological positional awareness to robust GRN inference.

### 2.6. Parameter Analysis

Most existing gene regulatory network inference methods are highly sensitive to hyperparameter settings [6] and often require extensive tuning to achieve optimal performance.

To evaluate the robustness of IAGRN and select a unified default parameter configuration, we conducted a systematic hyperparameter sensitivity analysis using the Non-specific ChIP-seq reference networks across the seven benchmark datasets. We focused on three key hyperparameters: (1) the dropout rate, (2) the shortest-path distance (SPD) bias weight, implemented as bias_weight, and (3) the number of interleaved encoder layers.

Candidate values for these hyperparameters were evaluated by varying one factor at a time while holding the other settings fixed. A single shared configuration, rather than a dataset-specific configuration, was selected based on validation performance and was subsequently fixed for all reference-network and gene-scale evaluations. Neither test-set AUROC nor test-set AUPRC was used for hyperparameter selection.

The tested parameter ranges were defined as follows: (1) dropout rate: 0, 0.1, 0.2, 0.3, and 0.4; (2) SPD bias weight: 0, 0.1, 0.2, 0.3, and 0.4; and (3) number of interleaved encoder layers: 1, 2, 3, 5, and 8. For each configuration, validation performance was quantified using AUROC and AUPRC under the same data-partitioning protocol described above. This analysis guided the selection of a unified default configuration and characterized the sensitivity of model performance to the evaluated hyperparameters.

**Figure 5:**
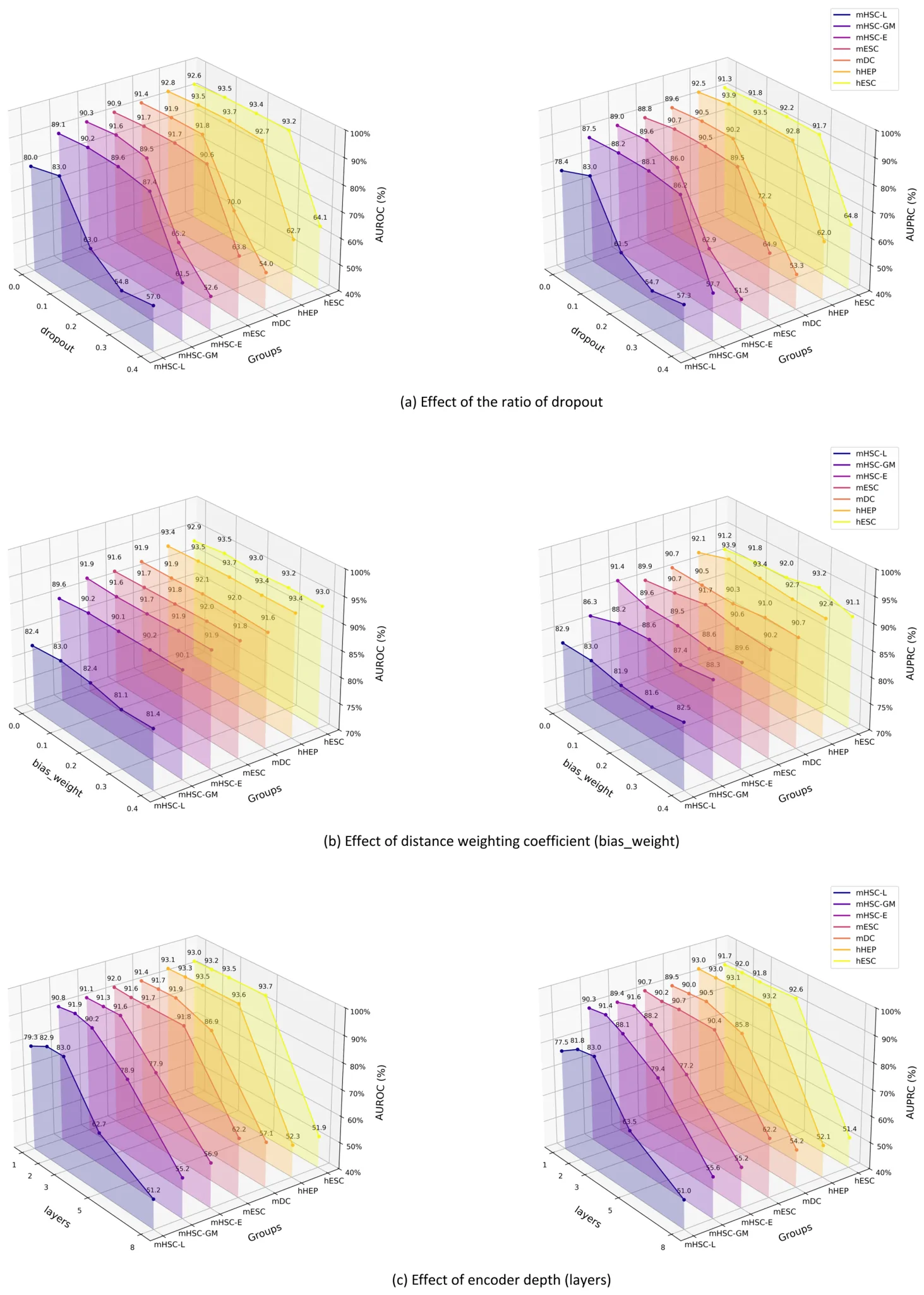
Parameter sensitivity analysis of IAGRN on seven benchmark datasets. The 3D waterfall plots illustrate the influence of three key hyperparameters on model performance, evaluated using AUROC (left column) and AUPRC (right column). The x-axis represents the hyperparameter values, the y-axis (depth direction) corresponds to different datasets, and the z-axis (height) indicates the performance scores (%).

The experimental results shown in Figure 5 indicate that the sensitivity of IAGRN differs across the three hyperparameters. Performance remains relatively stable under moderate dropout rates and SPD bias weights, whereas excessive encoder depth results in more evident performance degradation. Specifically, for the dropout rate, the experiments reveal that scRNA-seq data are highly sensitive to information loss. Although moderate regularization can improve generalization ability, when the dropout rate exceeds 0.2, the model performance on the sparse dataset mHSC-L drops sharply, indicating that excessive dropout disrupts already sparse biological signals and leads to underfitting. For the distance-based soft bias coefficient in global attention, the results show that introducing moderate structural guidance effectively filters global noise and improves performance. However, excessively large coefficients (greater than 0.2) impose overly strong penalties on long-range interactions, thereby limiting the model’s ability to capture long-distance regulatory dependencies. Regarding the number of stacked layers in the interleaved attention encoder, we observe that as the number of layers increases from one to three, the model performance steadily improves on most datasets, which can be attributed to the enhanced ability of deeper networks to extract higher-level semantic features. However, when the number of layers reaches eight, the performance collapses across all datasets; for example, the AUROC on mHSC-L decreases to 51.9%.

Within the evaluated ranges, a dropout rate of 0.1, an SPD bias weight of 0.1, and three interleaved encoder layers provided a favorable balance between validation performance and architectural complexity and were therefore adopted as the default configuration. The same configuration was subsequently applied across all datasets, reference-network types, and gene-scale settings without dataset-specific tuning. This shared configuration supports a consistent evaluation protocol, limits opportunities for dataset-specific hyperparameter optimization, and facilitates direct comparison across the evaluated settings.

### 2.7. Computational Efficiency and Scalability Considerations

To assess the practical applicability of IAGRN, we analyzed its computational characteristics from both theoretical and empirical perspectives. Let *N* denote the number of genes, $d_{\mathrm{model}}$ the hidden dimension, and *L* the number of encoder layers. For each encoder layer, dense attention computation requires $O(N^{2}d_{\mathrm{model}})$ time, while feature projection and feed-forward transformation require $O(Nd_{\mathrm{model}}^{2})$ time. The resulting encoder complexity is $O(LN^{2}d_{\mathrm{model}}+LNd_{\mathrm{model}}^{2})$, with the principal attention-matrix storage scaling as $O(N^{2})$ per layer. This quadratic term arises from the dense attention formulation used to model pairwise gene dependencies. Topology-masked attention and global self-attention share this computational formulation but employ different structural matrices to regulate information flow.

Several design choices reduce repeated computation outside the attention operation. The default encoder contains only three interleaved layers, while both Laplacian positional encoding and the shortest-path distance matrix are computed once during preprocessing and subsequently reused. The resulting all-pairs shortest-path distance matrix requires $O(N^{2})$ storage. For *P* candidate TF–TG pairs, the role-specific inner-product decoder requires $O(Pd_{\mathrm{out}})$ time. Consequently, the principal computational cost is concentrated in the attention operation, whereas structural preprocessing is amortized across model iterations and link prediction remains lightweight.

To ensure a consistent runtime comparison, all methods were evaluated on the mHSC-GM dataset under the same computational environment and the TFs+1000 gene-scale setting. Runtime was defined as the combined wall-clock time for model training and inference, with one-time structural preprocessing excluded. The values reported in Table 3 represent the mean runtimes over 20 independent runs for each reference-network type.

As shown in Table 3, IAGRN required 240.3 s with the Non-specific ChIP-seq reference network and 212.0 s with the STRING reference network under the TFs+1000 setting. In both cases, IAGRN achieved the third-lowest runtime among the six compared methods. With the Non-specific ChIP-seq reference network, its runtime was 38.2%, 10.8%, and 2.8% lower than those of GENIE3, GNNLink, and GRANet, respectively. With the STRING reference network, the corresponding reductions were 55.4%, 20.8%, and 4.1%. Although GNE and GENELink required less time, these results indicate that IAGRN maintains a competitive computational cost relative to the selected comparison methods.

Overall, the theoretical complexity and measured runtimes indicate that IAGRN maintains a competitive computational cost at the evaluated gene scale. When considered together with the predictive results, these measurements support a favorable performance–runtime trade-off rather than uniformly superior efficiency. The quadratic dependence on *N* arises from the current dense attention implementation and becomes an important consideration for substantially larger networks. Sparse or efficient attention mechanisms may further extend the applicable network scale while preserving the interleaved local–global architecture.

### 2.8. Case Study

To further evaluate the capability of IAGRN in identifying potential target genes of specific transcription factors, we conducted an in-depth case study based on the trained model. The analysis was performed on the Cell-type-specific ChIP-seq hESC TFs+1000 dataset.

Specifically, IAGRN was used to predict TF–TG interactions. To construct a biologically interpretable regulatory subnetwork, we restricted the number of predicted target genes for each TF to no more than 20 and extracted the top 100 predicted TF–TG interactions according to their ranking scores. From this subnetwork, we focused on two TFs with important biological relevance: TFAP2A and JUND.

TFAP2A is a key transcription factor involved in regulating cell proliferation, differentiation, and apoptosis. It is widely reported to function as a tumor suppressor in multiple cancer types, and mutations in TFAP2A are known to cause branchio-oculo-facial syndrome [34,35]. JUND, a core component of the AP-1 transcriptional complex, plays context-dependent regulatory roles and can either promote or suppress pathological processes. It is critically involved in regulating cellular stress responses and cell cycle progression [36].

**Figure 6:**
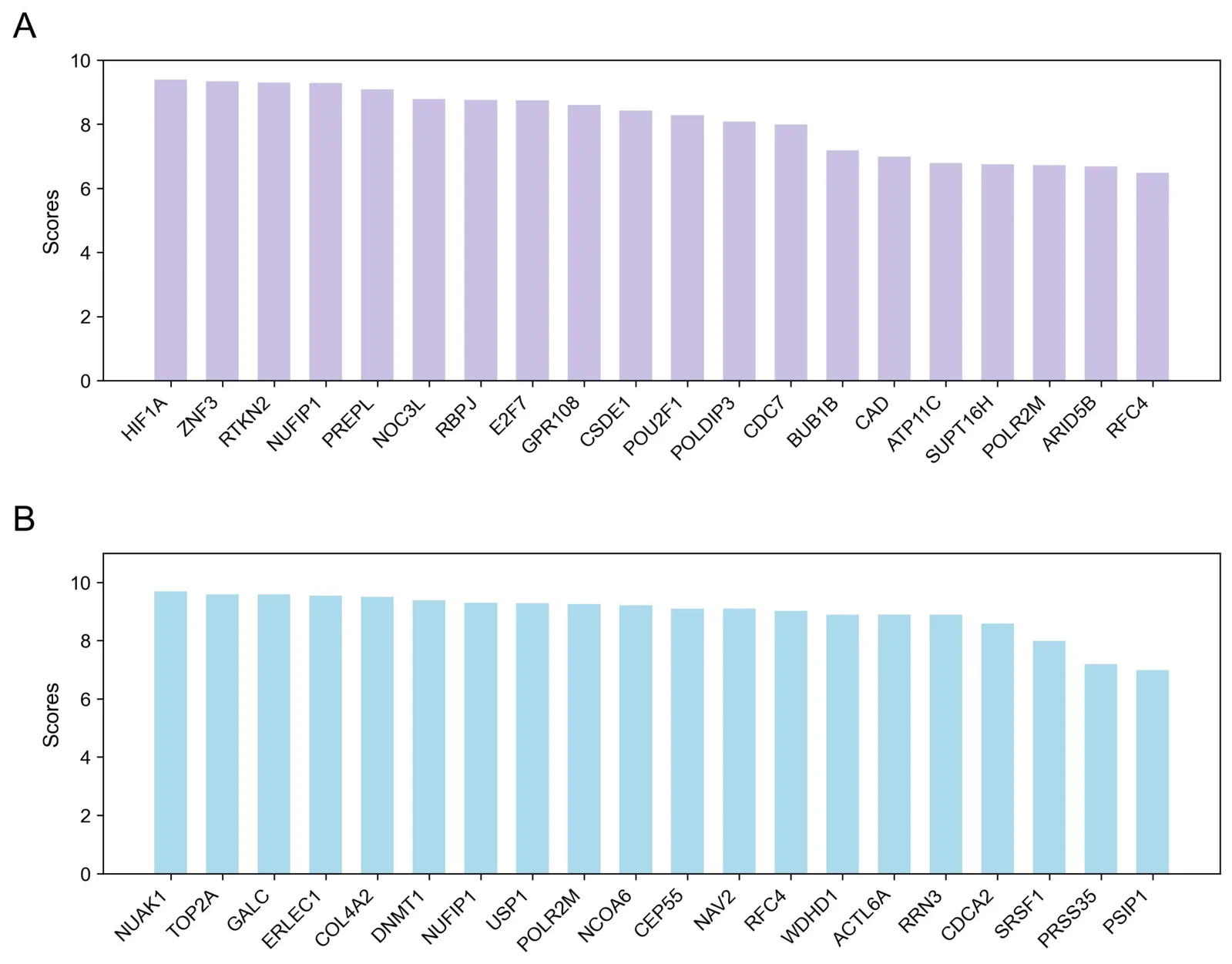
Application of IAGRN for predicting potential target genes in the hESC dataset. (**A**) Ranking plot of the top 20 predicted target gene scores for JUND. (**B**) Ranking plot of the top 20 predicted target gene scores for TFAP2A.

**Figure 7:**
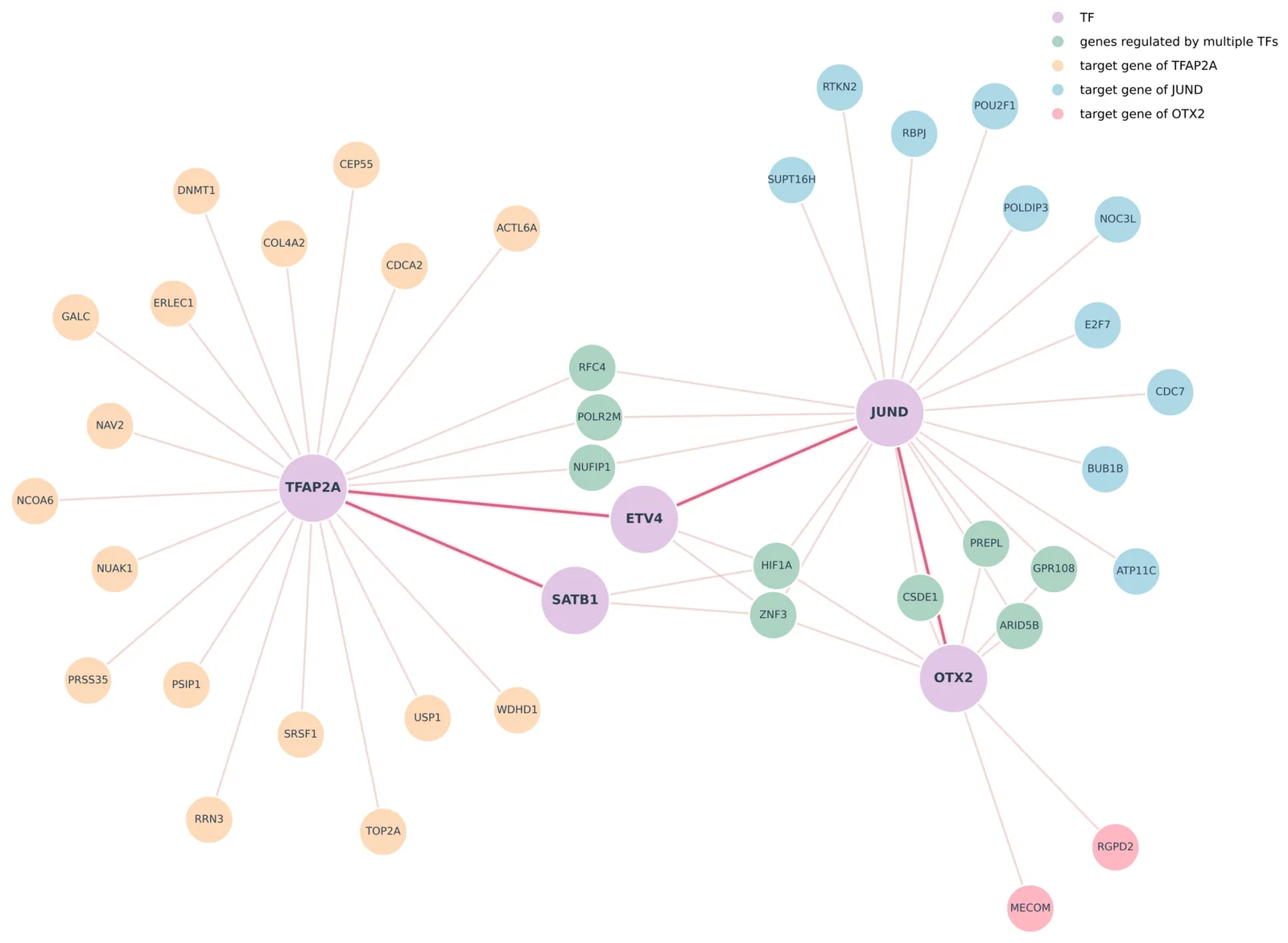
Visualization of the IAGRN-predicted gene regulatory network. Node colors represent the gene categories indicated in the legend. Light-colored edges represent predicted TF–target gene interactions, whereas relatively darker edges represent predicted interactions between transcription factors.

To evaluate predictive accuracy, we extracted the top 20 candidate target genes for JUND and TFAP2A based on their predicted regulatory scores (Figure 6). These predictions were subsequently cross-referenced with experimentally supported interactions in the hTFtarget database [37]. Remarkably, the validation confirmed 16 of the 20 predicted targets for TFAP2A, while all 20 targets for JUND were fully supported by experimental evidence. The detailed interaction profiles and the reconstructed regulatory subnetwork are presented in Table 4 and Figure 7, respectively.

Analysis of the visualized gene regulatory network reveals that our model successfully identifies indirect regulatory cascades that remain undetected by existing methods. For instance, as depicted in the network, the transcription factors JUND and TFAP2A achieve direct and indirect regulation of their downstream targets via intermediate regulators; specifically, JUND operates through ETV4 and OTX2, while TFAP2A orchestrates its cascade via ETV4 and SATB1. Notably, the absence of experimental validation for certain predicted target genes, particularly those regulated by TFAP2A, does not necessarily indicate incorrect predictions. Given that current regulatory databases and experimental resources remain incomplete, these interactions may correspond to context-specific or previously unexplored regulatory relationships that have yet to be characterized. Therefore, IAGRN provides not only accurate reconstruction of known TF–gene regulatory patterns but also a systematic approach for prioritizing promising candidate interactions beyond existing knowledge. Subsequent experimental investigations of these candidates may uncover novel regulatory circuits and contribute to a more comprehensive understanding of transcriptional regulation.

## 3. Discussion

Accurate reconstruction of GRNs is a fundamental task for elucidating cellular heterogeneity, understanding complex disease mechanisms, and identifying potential therapeutic targets. IAGRN is designed to balance prior structural constraints with the modeling of regulatory dependencies beyond observed local neighborhoods. By interleaving topology-masked attention and global self-attention, the framework combines hard local constraints with soft global structural guidance within a unified encoder. The ablation results support the complementary contributions of these components, indicating that their coordinated use, rather than either mechanism alone, contributes to predictive performance.

The hESC case study further illustrates the potential biological utility of the framework through the TFAP2A-centered subnetwork. Sixteen of the twenty highest-ranked TFAP2A targets were supported by hTFtarget. Given the association between pathogenic TFAP2A variants and branchio-oculo-facial syndrome (BOFS), these predictions provide a hypothesis-generating set of regulatory interactions relevant to craniofacial development. However, because the data were obtained from hESCs rather than disease-specific tissues, this subnetwork should not be interpreted as a patient-specific BOFS network and requires validation in relevant cellular or perturbation-based models.

Furthermore, the incorporation of LapPE effectively compensates for the lack of topological awareness in standard global attention mechanisms. Across the seven benchmark datasets and the evaluated network settings, IAGRN achieved consistent and competitive performance, with an average relative AUROC improvement of approximately 4.5% over the second-best methods. The ablation analyses suggest that integrating interleaved attention with LapPE helps preserve structural distinctions among genes while extending information exchange beyond conventional local receptive fields. Nevertheless, these findings are specific to the datasets and evaluation protocols examined in this study and do not imply uniform superiority across all GRN inference settings.

The training-derived graph provides an informative structural inductive bias, but its quality may influence model performance. Although held-out interactions are excluded from graph construction, missing or spurious training edges may affect local aggregation and the structural encodings derived from the graph. Global self-attention enables information exchange beyond observed neighborhoods and may reduce strict dependence on individual local connections, but it cannot completely remove the influence of prior-network quality. Further evaluation using systematically perturbed and independently constructed priors would provide a more comprehensive assessment of this sensitivity.

Computationally, the dense encoder has a time complexity of $O(LN^{2}d_{\mathrm{model}}+LNd_{\mathrm{model}}^{2})$ and principal attention-matrix storage of $O(N^{2})$ per layer. Shortest-path distances and LapPE are computed once and reused, while link scoring requires only $O(Pd_{\mathrm{out}})$ time for *P* candidate TF–TG pairs. Under the TFs+1000 setting, IAGRN exhibited the third-lowest runtime among the six methods in Table 3, supporting a competitive balance between predictive performance and computational cost rather than uniformly superior efficiency. The quadratic dependence on the number of genes nevertheless limits direct extension to substantially larger GRNs. Sparse attention and more efficient structural encodings may improve scalability in future implementations.

Finally, the current formulation reconstructs static GRNs and does not explicitly model regulatory rewiring over time or pseudotime. Its node-transductive evaluation also does not establish transfer to independent cohorts, platforms, or previously unseen gene sets. Future work will therefore explore temporal modeling, multi-omic integration, independent-cohort validation, and scalable attention mechanisms to extend the framework beyond its current application scope.

## 4. Materials and Methods

**Figure 8:**
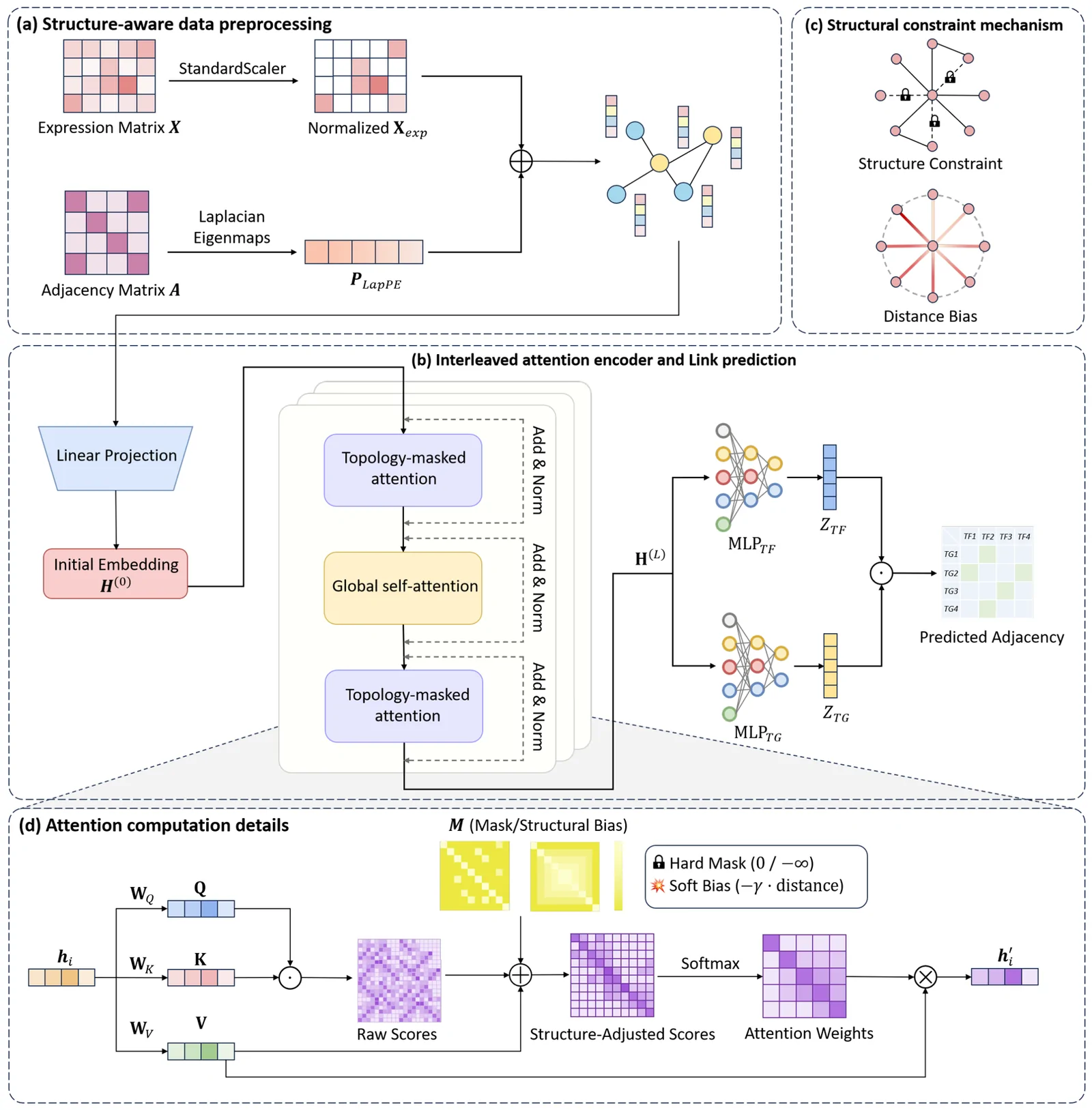
Overview of the IAGRN framework. (**a**) Normalized gene expression and Laplacian positional encodings are concatenated and mapped via a linear projection to generate initial embeddings. (**b**) The encoder alternates between masked attention and global self-attention blocks. Final representations are decoupled into TF and Target embeddings via dual MLPs for dot-product scoring. (**c**) Topology-masked attention applies hard masking for local inductive bias, while global self-attention incorporates distance-based soft bias for global receptive fields. (**d**) The left and right yellow matrices illustrate the layer-specific forms of the unified structural matrix $M^{\left(l\right)}$. In a topology-masked attention layer, $M^{\left(l\right)}$ acts as a hard topology mask, with $M_{ij}^{\left(l\right)}=0$ for an allowed neighbor or self-interaction and −∞ otherwise. In a global self-attention layer, $M^{\left(l\right)}$ acts as a soft distance bias, defined as $M_{ij}^{\left(l\right)}=-\gamma d_{\mathrm{SPD}}\left(v_{i},v_{j}\right)$. At each encoder layer, the corresponding form of $M^{\left(l\right)}$ is added to the scaled dot-product attention logits before the softmax operation.

According to the characteristics of GRN inference, this task can be formulated as a link prediction problem [14,38]. The proposed IAGRN framework aims to reconstruct potential regulatory relationships between TFs and TGs from high-dimensional and sparse scRNA-seq data. The overall architecture of the model is illustrated in Figure 8.

### 4.1. Structure-Aware Data Preprocessing

The input to the model consists of two components: a single-cell gene expression matrix $X\in R^{N\times M}$, where *N* denotes the number of genes and *M* denotes the number of cells, and a gene adjacency matrix $A\in R^{N\times N}$ representing the training graph, constructed exclusively from positive regulatory edges in the training partition. The complete benchmark reference network is partitioned before the construction of any topology-dependent model input. Consequently, all structural information supplied to the encoder is derived solely from A, ensuring that held-out positive edges cannot influence representation learning through the graph topology. Negative sampled gene pairs are used only as labeled instances for model supervision or evaluation and do not alter the encoder graph. Raw scRNA-seq data are typically influenced by variations in sequencing depth and technical noise, and are characterized by high sparsity and skewed distributions. To mitigate the impact of differences in gene expression scales on model training, we apply Z-score normalization to the expression values of each gene across all cells [39,40], such that the mean is 0 and the standard deviation is 1. Specifically, a linear transformation is applied to the expression values. For the expression level $e_{ij}$ of gene *i* in cell *j*, the normalization process is defined as follows:

(2) $$x_{ij}=\frac{e_{ij}-\mu_{i}}{\sigma_{i}}.$$

Here, $\mu_{i}$ and $\sigma_{i}$ denote the mean and standard deviation of the expression values of gene *i* across all cells, respectively. After normalization, we obtain the standardized expression feature matrix $X_{\exp}\in R^{N\times M}$.

The subsequently introduced global self-attention mechanism compensates for the limitations of traditional GNNs in capturing node positional information. To address this issue, it is necessary to explicitly incorporate the topological structure of the graph into node features. Specifically, the eigenvectors of the graph Laplacian matrix are adopted as positional encoding (LapPE) [41]. The symmetric normalized graph Laplacian is defined as:

(3) $$L=I-D^{-\frac{1}{2}}AD^{-\frac{1}{2}},$$

where I is the identity matrix and D is the degree matrix with diagonal entries defined as $D_{ii}=\sum_{j}A_{ij}$.

We then perform eigendecomposition on L:

(4) $$Lu_{k}=\lambda_{k}u_{k},$$

where $\lambda_{k}$ and $u_{k}$ denote the *k*-th eigenvalue and its corresponding eigenvector, respectively. According to spectral graph theory, eigenvectors associated with smaller eigenvalues encode smooth structural information, whereas larger eigenvalues correspond to high-frequency components that capture local variations.

To incorporate informative structural context, we select the eigenvectors corresponding to the *K* smallest non-trivial eigenvalues to construct the topological positional encoding matrix $P_{\mathrm{LapPE}}\in R^{N\times K}$:

(5) $$P_{\mathrm{LapPE}}=\left[u_{1},u_{2},\ldots ,u_{K}\right].$$

These eigenvectors serve as structural coordinates of genes within the regulatory network, effectively encoding low-frequency structural information that reflects clustering behavior and relative node positions.

Finally, the normalized gene expression features $X_{\exp}$ are concatenated with the Laplacian positional encodings $P_{\mathrm{LapPE}}$ along the feature dimension to form the final input feature matrix:

(6) $$H_{\mathrm{in}}^{\left(0\right)}=\mathrm{Concat}\left(X_{\exp},P_{\mathrm{LapPE}}\right)\in R^{N\times (M+K)}.$$

This fused representation integrates transcriptomic signals reflecting gene functional activity with structural coordinates capturing regulatory roles, thereby providing comprehensive information for the subsequent interleaved attention encoder.

### 4.2. Latent Feature Projection

After the aforementioned preprocessing, directly feeding the raw input matrix into the subsequent attention encoder introduces two primary challenges. First, gene expression features and Laplacian positional encodings follow fundamentally different statistical distributions; naive interactions between them may hinder optimization and destabilize training [42]. Second, the original feature dimensionality $d_{\mathrm{in}}=M+K$ is typically high and varies across datasets, necessitating projection into a fixed, lower-dimensional hidden space $d_{\mathrm{model}}$ to reduce computational complexity and facilitate higher-level semantic abstraction.

The input feature matrix obtained after preprocessing is denoted as $H_{\mathrm{in}}^{\left(0\right)}\in R^{N\times d_{\mathrm{in}}}$, which integrates gene expression features and Laplacian positional encodings. Here, *N* represents the number of genes and $d_{\mathrm{in}}$ denotes the total input feature dimension. To capture higher-order interactions among heterogeneous features and align the representation with the internal hidden dimension $d_{\mathrm{model}}$, a learnable linear projection is applied. This operation not only performs dimensionality reduction but also enables an initial weighted fusion of expression and topological features through a trainable weight matrix. For each gene node *i*, the projection is computed as:

(7) $$z_{i}=\left[x_{i}\|p_{i}\right]W_{\mathrm{proj}}+b_{\mathrm{proj}},$$

where $\left[x_{i}\|p_{i}\right]\in R^{M+K}$ denotes the concatenated input vector of gene *i*, $W_{\mathrm{proj}}\in R^{d_{\mathrm{in}}\times d_{\mathrm{model}}}$ is a learnable projection matrix, and $b_{\mathrm{proj}}\in R^{d_{\mathrm{model}}}$ is the bias vector.

To accelerate convergence and mitigate vanishing and exploding gradients, layer normalization is applied after the linear transformation. Specifically, normalization is performed over each node-wise feature vector to ensure zero mean and unit variance before entering the attention module:

(8) $${\hat{z}}_{i}=\mathrm{LayerNorm}\left(z_{i}\right)=\frac{z_{i}-\mu_{i}}{\sqrt{\sigma_{i}^{2}+\epsilon }}⊙\gamma +\beta ,$$

where $\mu_{i}$ and $\sigma_{i}$ denote the mean and standard deviation of $z_{i}$, respectively, ϵ is a small constant for numerical stability, and γ and β are learnable affine transformation parameters.

Subsequently, a LeakyReLU activation function [43] is employed to introduce nonlinearity. To prevent overfitting and improve generalization capability, a dropout mechanism [44] is further applied. The resulting initial node embedding matrix $H^{\left(0\right)}\in R^{N\times d_{\mathrm{model}}}$ is given by:

(9) $$H^{\left(0\right)}=\mathrm{Dropout}\left(\mathrm{LeakyReLU}\left(\hat{Z}\right)\right).$$

After these transformations, the matrix $H^{\left(0\right)}\in R^{N\times d_{\mathrm{model}}}$ serves as the initial input state of the encoder. This embedding effectively integrates heterogeneous transcriptomic signals and structural positional information into a unified latent manifold [45], thereby facilitating efficient interaction within the subsequent interleaved attention mechanism.

### 4.3. Interleaved Attention Encoder

To address the sparsity of scRNA-seq data [5] and to balance the inductive bias of prior structures with the exploration of global contextual information, we propose an interleaved attention encoder composed of topology-masked attention and global self-attention. The core design principle of the encoder is interleaved local–global feature extraction. Specifically, the encoder is built by stacking *l* layers of node set attention blocks (NSABs), which take the initial embeddings $H^{\left(0\right)}$ from the input projection module as input. To effectively integrate multi-level biological information, an interleaving strategy is employed during stacking, systematically alternating between topology-masked attention and global self-attention to capture complementary structural and contextual information. In this study, a three-layer interleaved architecture of topology-masked attention→global self-attention→topology-masked attention is adopted as the default configuration, aiming to first stabilize features through local constraints, then expand contextual information via global attention, and finally refine representations through local aggregation.

#### 4.3.1. Node Set Attention Block

The node set attention block serves as the fundamental building block of the encoder. Each NSAB consists of two sublayers: a multi-head attention (MHA) layer and a position-wise feed-forward network (FFN). To facilitate deep gradient propagation and accelerate convergence, residual connections [46] and layer normalization [42] are applied around each sublayer.

Let $H^{\left(l-1\right)}\in R^{N\times d_{\mathrm{model}}}$ denote the input feature matrix of the *l*-th layer, where *N* is the number of genes and $d_{\mathrm{model}}$ is the feature dimension. The computation proceeds as follows.

First, information is aggregated via the multi-head attention mechanism:

(10) $${\hat{H}}^{\left(l\right)}=\mathrm{LayerNorm}\left(H^{\left(l-1\right)}+\mathrm{Dropout}\left(\mathrm{MHA}(H^{\left(l-1\right)};M^{\left(l\right)})\right)\right).$$

Then, feature transformation is performed through the feed-forward network:

(11) $$H^{\left(l\right)}=\mathrm{LayerNorm}\left({\hat{H}}^{\left(l\right)}+\mathrm{Dropout}\left(\mathrm{FFN}\left({\hat{H}}^{\left(l\right)}\right)\right)\right),$$

where $M^{\left(l\right)}$ denotes the attention mask or bias matrix at layer *l*, depending on whether the layer corresponds to a masked attention block or a global self-attention block.

#### 4.3.2. Generalized Multi-Head Attention

To unify the topology-masked attention and the structure-aware global self-attention under a single formulation, we introduce a generalized multi-head attention mechanism augmented with structural bias. This formulation extends the scaled dot-product attention by incorporating graph-dependent bias terms into the attention logits. Given the input feature matrix at layer l−1:

(12) $$H^{\left(l-1\right)}\in R^{N\times d_{\mathrm{model}}},$$

where *N* denotes the number of genes and $d_{\mathrm{model}}$ is the embedding dimension, we project the input into *h* parallel attention heads [47]. For the *k*-th head (k=1,…,h):

(13) $$Q_{k}=H^{\left(l-1\right)}W_{Q}^{k},$$

(14) $$K_{k}=H^{\left(l-1\right)}W_{K}^{k},$$

(15) $$V_{k}=H^{\left(l-1\right)}W_{V}^{k},$$

where

(16) $$W_{Q}^{k},W_{K}^{k},W_{V}^{k}\in R^{d_{\mathrm{model}}\times d_{k}},$$

and $d_{k}=d_{\mathrm{model}}/h$ is the dimensionality of each attention head. The scaled dot-product attention with structural bias is defined as:

(17) $${\mathrm{Head}}_{k}=\mathrm{softmax}\left(\frac{Q_{k}K_{k}^{⊤}}{\sqrt{d_{k}}}+M^{\left(l\right)}\right)V_{k}.$$

Here, $M^{\left(l\right)}\in R^{N\times N}$ is a structural bias matrix added directly to the attention logits to regulate information flow [48]. Within each layer, the same structural bias matrix is shared across attention heads to maintain a consistent structural prior while allowing feature subspace specialization. In practice, masking is approximated using large negative constants for numerical stability.

Finally, the outputs of all heads are concatenated and linearly transformed:

(18) $$\mathrm{MHA}\left(H^{\left(l-1\right)}\right)=\left[{\mathrm{Head}}_{1}\;∥\;\ldots \;∥\;{\mathrm{Head}}_{h}\right]W_{O},$$

where $W_{O}$ is a learnable output projection matrix.

This generalized formulation enables flexible instantiation of different attention behaviors through different definitions of $M^{\left(l\right)}$.

#### 4.3.3. Structure-Aware Dual Attention Strategies

To balance structural inductive bias and global expressive capacity, we design two complementary attention modes by defining different structural bias matrices $M^{\left(l\right)}$ at each layer.

(1) Topology-masked attention:

This mode enforces strong local structural constraints by leveraging the prior gene regulatory network. It restricts each node to aggregate information only from its neighbors, similar to message passing in graph convolutional networks, while maintaining dynamic attention weights. In this case, $M^{\left(l\right)}$ is defined as a hard mask derived from the prior adjacency matrix A:

(19) $$M_{ij}^{\left(l\right)}=\left\{\begin{array}{cc} 0, & \mathrm{if}\;A_{ij}=1\;\mathrm{or}\;i=j,\\ -\infty , & \mathrm{otherwise}. \end{array}\right.$$

This formulation forces the attention scores between non-adjacent nodes to −∞, resulting in zero attention weights after the softmax operation and ensuring precise modeling of local topology.

Topology-masked attention shares with graph attention networks (GATs) the restriction of aggregating information from graph neighbors but differs in its computational formulation and architectural role. A standard GAT layer performs additive attention over projected neighboring features as a message-passing operator. In contrast, IAGRN represents the prior graph as an additive hard mask within Transformer-style scaled dot-product multi-head attention and embeds this operation in a block containing residual connections, layer normalization, and a feed-forward network. These topology-masked blocks are further interleaved with global self-attention blocks incorporating a soft distance-based bias. Thus, although neighborhood masking itself is a familiar structural inductive bias, the distinctive design of IAGRN lies in alternating hard local constraints and soft global guidance within a unified encoder.

(2) Global self-attention:

To capture long-range dependencies, this mode removes local restrictions and enables global node interactions. To preserve topological awareness during global attention, we introduce a soft structural bias based on the shortest path distance (SPD) [49]. The structural bias matrix is defined as:

(20) $$M_{ij}^{\left(l\right)}=-\gamma \;d_{\mathrm{SPD}}\left(v_{i},v_{j}\right),$$

where $d_{\mathrm{SPD}}\left(v_{i},v_{j}\right)$ denotes the shortest-path distance between nodes $v_{i}$ and $v_{j}$ in the training-derived graph, and γ>0 is a tunable hyperparameter controlling the strength of the distance-based structural bias. Based on validation performance, γ was set to 0.1 and subsequently fixed for all reported experiments.

This mechanism enables the model to maintain a global receptive field while adaptively emphasizing topologically closer nodes, thereby imposing a soft structural constraint during global interactions.

### 4.4. Link Prediction

After *L* layers of the interleaved attention encoder, we obtain the final node embedding matrix $H^{\left(L\right)}\in R^{N\times d_{\mathrm{model}}}$, which encodes rich structural and semantic information.

To model the inherent directionality of gene regulatory interactions, we employ two independent MLP branches to project the shared node embeddings into role-specific representations for TFs and TGs, respectively.

For a gene node *i* with embedding $h_{i}^{\left(L\right)}$, we first compute the hidden representations:

(21) $$g_{\mathrm{TF},i}=\sigma \;\left(W_{\mathrm{TF}}^{\left(1\right)}h_{i}^{\left(L\right)}+b_{\mathrm{TF}}^{\left(1\right)}\right),$$

(22) $$g_{\mathrm{TG},i}=\sigma \;\left(W_{\mathrm{TG}}^{\left(1\right)}h_{i}^{\left(L\right)}+b_{\mathrm{TG}}^{\left(1\right)}\right).$$

After dropout regularization, the role-specific embeddings are obtained as:

(23) $$Z_{\mathrm{TF},i}=\sigma \;\left(W_{\mathrm{TF}}^{\left(2\right)}\;\mathrm{Dropout}\left(g_{\mathrm{TF},i}\right)+b_{\mathrm{TF}}^{\left(2\right)}\right),$$

(24) $$Z_{\mathrm{TG},i}=\sigma \;\left(W_{\mathrm{TG}}^{\left(2\right)}\;\mathrm{Dropout}\left(g_{\mathrm{TG},i}\right)+b_{\mathrm{TG}}^{\left(2\right)}\right),$$

where $W^{\left(1\right)},W^{\left(2\right)}$ and $b^{\left(1\right)},b^{\left(2\right)}$ denote the learnable weight matrices and bias vectors of each layer, and σ(·) represents a nonlinear activation function.

The resulting vectors $Z_{\mathrm{TF},i}$ and $Z_{\mathrm{TG},i}$ characterize gene *i* under the TF and TG roles, respectively.

To predict whether a regulatory interaction exists from transcription factor *u* to target gene *v*, we compute a matching score using the inner product of their role-specific embeddings:

(25) $$s_{uv}=Z_{\mathrm{TF},u}^{⊤}Z_{\mathrm{TG},v}=\sum_{k=1}^{d_{\mathrm{out}}}{\left(Z_{\mathrm{TF},u}\right)}_{k}{\left(Z_{\mathrm{TG},v}\right)}_{k}.$$

Here, $d_{\mathrm{out}}$ denotes the dimensionality of the role-specific TF and TG embeddings; $d_{\mathrm{out}}$ was set to 16 in all experiments.

The predicted probability is obtained via a sigmoid function:

(26) $${\hat{y}}_{uv}=\sigma \left(s_{uv}\right).$$

During training, we optimize the model parameters using the binary cross-entropy (BCE) loss.

Given a training set D containing gene pairs (u,v) with ground-truth labels $y_{uv}\in \left\{0,1\right\}$, the loss function is defined as:

(27) $$L=-\frac{1}{\left|D\right|}\sum_{(u,v)\in D}\left[y_{uv}\log{\hat{y}}_{uv}+\left(1-y_{uv}\right)\log\left(1-{\hat{y}}_{uv}\right)\right].$$

By minimizing this objective, the model learns discriminative role-aware gene representations that effectively distinguish regulatory from non-regulatory gene pairs.

## 5. Conclusions

In this work, we propose IAGRN, an interleaved-attention graph neural network framework for GRN inference from highly sparse single-cell RNA sequencing data. By integrating training-derived structural information with global regulatory dependencies through a generalized structure-aware attention mechanism, IAGRN is designed to model both local regulatory patterns and long-range gene interactions that may be difficult to identify using conventional message-passing approaches. Evaluations across seven benchmark datasets showed that IAGRN achieved consistent and competitive performance under the examined reference-network and gene-scale settings, while reducing its reliance on strictly local information aggregation. These findings suggest that IAGRN provides a useful computational framework for investigating regulatory relationships in complex cellular systems. The inferred networks may also support hypothesis generation and the prioritization of key regulatory factors and candidate disease-relevant interactions, although such applications require further validation using disease-specific data and biological experiments.

Future work will focus on further improving the biological interpretability and applicability of IAGRN. First, we plan to incorporate multi-omics information, such as scATAC-seq-derived chromatin accessibility signals, to dynamically update regulatory priors and enhance the biological context of inferred GRNs. Second, we aim to extend the current framework with temporal modeling strategies to reconstruct dynamic, time-resolved GRNs, with the aim of better characterizing regulatory changes associated with cellular differentiation and developmental trajectories.

## Figures and Tables

**Table 1:** Details of datasets used in this work.

Dataset	Species	Nodes	STRING	Non-Specific ChIP-seq	Cell-Type-Specific ChIP-seq
hESC	Human	910 (1410)	4257 (5149)	3441 (4617)	4545 (7084)
hHEP	Human	948 (1448)	7523 (9003)	4129 (5351)	9939 (15,558)
mDC	Mouse	821 (1321)	4815 (5898)	3067 (3918)	756 (1193)
mESC	Mouse	1120 (1620)	7762 (8479)	6893 (8030)	29,613 (42,795)
mHSC-E	Mouse	704 (1204)	1371 (1826)	1425 (1960)	11,557 (21,975)
mHSC-GM	Mouse	632 (1132)	748 (1311)	743 (1358)	7364 (14,135)
mHSC-L	Mouse	560 (692)	137 (154)	279 (317)	4398 (5180)

*Note:* Values outside parentheses correspond to the TFs+500 setting, whereas values inside parentheses correspond to the TFs+1000 setting. TFs+500 and TFs+1000 include all retained TFs together with 500 and 1000 highly variable non-TF genes, respectively.

**Table 2:** Variants of IAGRN and their modifications.

Model Variant	TMA	GSA	Interleaved	LapPE
IAGRN	✓	✓	✓	✓
w/o GSA	✓	–	–	✓
w/o TMA	–	✓	–	✓
w/o Interleaved	✓	✓	–	✓
w/o LapPE	✓	✓	✓	–

*Note:* ✓ indicates that the component is retained, whereas an en dash (–) indicates that the component is removed.

**Table 3:** Mean training-and-inference runtime (s) of different GRN inference methods on the mHSC-GM dataset across reference-network types under the TFs+1000 gene-scale setting. Structural preprocessing was excluded from the reported runtime.

Network Type	Gene Scale	GENIE3	GNE	GENELink	GNNLink	GRANet	IAGRN
Non-specific ChIP-seq	TFs+1000	389.0	216.1	209.0	269.4	247.1	240.3
STRING	TFs+1000	475.8	196.7	170.9	267.6	221.0	212.0

**Table 4:** Top 20 predicted target genes of JUND and TFAP2A.

Target Gene of JUND	Validation	Target Gene of TFAP2A	Validation
*HIF1A*	Validated by hTFtarget	*NUAK1*	Validated by hTFtarget
*ZNF3*	Validated by hTFtarget	*TOP2A*	Validated by hTFtarget
*RTKN2*	Validated by hTFtarget	*GALC*	Validated by hTFtarget
*NUFIP1*	Validated by hTFtarget	*ERLEC1*	Unconfirmed
*PREPL*	Validated by hTFtarget	*COL4A2*	Validated by hTFtarget
*NOC3L*	Validated by hTFtarget	*DNMT1*	Validated by hTFtarget
*RBPJ*	Validated by hTFtarget	*NUFIP1*	Unconfirmed
*E2F7*	Validated by hTFtarget	*USP1*	Validated by hTFtarget
*GPR108*	Validated by hTFtarget	*POLR2M*	Validated by hTFtarget
*CSDE1*	Validated by hTFtarget	*NCOA6*	Validated by hTFtarget
*POU2F1*	Validated by hTFtarget	*CEP55*	Validated by hTFtarget
*POLDIP3*	Validated by hTFtarget	*NAV2*	Validated by hTFtarget
*CDC7*	Validated by hTFtarget	*RFC4*	Validated by hTFtarget
*BUB1B*	Validated by hTFtarget	*WDHD1*	Unconfirmed
*CAD*	Validated by hTFtarget	*ACTL6A*	Unconfirmed
*ATP11C*	Validated by hTFtarget	*RRN3*	Validated by hTFtarget
*SUPT16H*	Validated by hTFtarget	*CDCA2*	Validated by hTFtarget
*POLR2M*	Validated by hTFtarget	*SRSF1*	Validated by hTFtarget
*ARID5B*	Validated by hTFtarget	*PRSS35*	Validated by hTFtarget
*RFC4*	Validated by hTFtarget	*PSIP1*	Validated by hTFtarget

## Data Availability

The original experimental datasets analyzed in this study are openly available on Zenodo at https://doi.org/10.5281/zenodo.3378975. The source code for implementing the proposed IAGRN framework is publicly available on GitHub at https://github.com/wangyue777/IAGRN (accessed on 9 August 2026).

## Abbreviations

GRN	Gene Regulatory Network
NSAB	Node Set Attention Block
TF	Transcription Factor
TG	Target Gene
